# Pregnant women as a reference population to estimate dengue under-reporting: methodological proposal and application in São Paulo State, Brazil

**DOI:** 10.1590/1980-549720260023

**Published:** 2026-07-27

**Authors:** Leandro Cesar Pompeu, Fredi Alexander Diaz-Quijano

**Affiliations:** I Universidade de São Paulo, Public Health School, Postgraduate Programme in Public Health Entomology – São Paulo (SP), Brazil.; II Universidade de São Paulo, Public Health School, Department of Epidemiology, Laboratory of Causal Inference in Epidemiology – São Paulo (SP), Brazil.

**Keywords:** Dengue, Under-reporting, Epidemiological monitoring, Pregnant women, Public health

## Abstract

**Objective:**

To propose and apply a methodology to estimate the under-reporting of probable dengue cases among non-pregnant women, using the incidence in pregnant women as a reference.

**Methods:**

Ecological study using data on probable dengue cases reported in the state of São Paulo, Brazil, between January and June 2024. Assuming that pregnant women have a higher likelihood of diagnosis and notification due to prenatal follow-up, this group was taken as a reference to calculate the under-reporting of dengue incidence among non-pregnant women aged 18 to 45 years. The association between under-reporting and factors such as Family Health Strategy coverage, number of Primary Health Care Units per thousand women at reproductive age, and degree of urbanization was assessed using logistic regression. Spatial autocorrelation analysis of under-reporting was also performed.

**Results:**

Of the 645 municipalities analyzed, 108 (16.7%) showed evidence of under-reporting, ranging from 1% to 95%. It was estimated that 8,624 dengue cases were not detected among non-pregnant women during the study period. No statistically significant associations were found between the analyzed factors and under-reporting. Spatial analysis identified significant clusters of municipalities with underreporting in the northwestern region of the state.

**Conclusion:**

The proposed methodology may identify areas with probable underreporting and support analyses of determinants of surveillance system sensitivity, thereby helping to guide interventions. Although potentially applicable to other notifiable diseases, comparisons between pregnant and non-pregnant women should be interpreted with caution due to differences in risk, vulnerability, and diagnostic criteria.

## INTRODUCTION

Dengue is the most important arboviral disease in the world. In 2024, Brazil recorded a record incidence, with 6,636,763 probable cases reported up to December of that year^1^. Moreover, the under-reporting of cases is expected in dengue surveillance, mainly due to the high proportion of mild or asymptomatic infections, estimated at about 75% of cases^2^. Although it is a recognized global problem, under-reporting remains difficult to identify and quantify^3^, potentially leading to an underestimation of the true disease burden and hindering control and prevention efforts^4^.

Pregnant women are among the groups at risk for severe dengue, and studies indicate an association with increased risk of preterm birth and fetal death^5,6^. Therefore, it is reasonable to assume that care for pregnant women has higher sensitivity in disease detection, supported by factors such as frequent antenatal follow-up, specialized care and more rigorous monitoring routines^7,8,9^. Thus, we hypothesize that the estimated incidence of cases in pregnant women more closely reflects the true incidence of the disease, serving as a reference to estimate under-reporting in other population groups with similar risk.

Although there are several strategies to estimate under-reporting, including active case finding in health services, population surveys and projections based on indirect data^10,11^, we found no studies exploring the use of specific groups with higher case-detection sensitivity as a reference for this estimation. Using such groups could facilitate large-scale quantification of under-reporting, allowing identification of clustering patterns and analysis of the influence of their determinants.

Consequently, we propose and apply a methodology to estimate under-reporting in non-pregnant women using the incidence of dengue in pregnant women as a reference. In doing so, we identified municipalities with possible under-reporting and assessed potentially associated factors. This method could help implement and monitor strategies to improve the sensitivity of the epidemiological surveillance system.

## METHODS

This is an ecological study using public, anonymized data from federal and state systems on notifications of probable dengue cases (PC), defined as reported cases minus discarded cases, in women from the municipalities of the State of São Paulo (ESP) between January and June 2024. The data were extracted in January 2025.

Although the age range usually considered childbearing age is 10-49 years^12^, in this study we analyzed only the incidences in pregnant and non-pregnant women aged 18-45 years. This age range was chosen to ensure comparability between groups in terms of infection risk and health-seeking behavior, and to exclude more extreme age groups in which differences in exposure, diagnosis and care received may occur. Non-pregnant women under 18 years of age display distinct patterns of health-service use, generally with lower care-seeking^13,14,15,16^, whereas pregnant adolescents typically receive intensified antenatal follow-up^13,14,17,18^. This restriction aimed to minimize selection bias and make the groups more homogeneous regarding viral exposure and surveillance sensitivity for case detection.

### Data source

Probable dengue cases were extracted from the Notifiable Health Conditions Information System (Sinan), available from the Department of Information and Informatics of the Unified Health System (DataSUS), and tabulated using Tabwin software, version 4.15^1^. These data included pregnancy status as one of the variables systematically collected, which allowed for numerators to calculate incidences in pregnant and non-pregnant women.

The denominators used to calculate incidence rates were estimated from a projection of the number of live births per municipality for the period, which were obtained from the Live Birth Information System (Sinasc), available on the Brazilian Open Data Portal^19^.

Population data and the variables urbanization level and number of Primary Health Units (UBS) per municipality, updated to June 2024, were extracted from the Tabnet system of the São Paulo State Health Secretariat (SES-SP) and from the 2023 estimates of the State Data Analysis System (Seade), based on the 2022 Census^20^. Data on Family Health Strategy (ESF) coverage were obtained from the e-Gestor Basic Care website of the Ministry of Health (MS), based on potential coverage which includes teams funded with federal, state and municipal resources^21^.

### Assumptions

For this study, we used the following assumptions for the proposed method:


(1) $$I_{p}=I_{np}|city,age$$

This equation refers to the assumption that the incidence of dengue in pregnant women *(I_P_)* is equal to the incidence in non-pregnant women (*I_np_*), conditional on factors such as municipality and age. Regarding the latter variable, we assume that restricting the study population provides sufficient conditioning, as previously mentioned.


(2) $$D_{p}\geq D_{np}|city,age$$

In this equation, we assume that the detected incidence in pregnant women (*D_p_*) will be equal to or greater than the detected incidence in non-pregnant women (*D_np_*), when controlled for municipality and age, because pregnant women tend to have greater contact with the health system, making it more likely that mild or moderate dengue cases are detected and reported. Therefore, *D_P_* ≥ *D_np_* is expected even ifthe true incidence is the same in both groups. From the above, we consider that in situations where *D_p_* < *D_np_* under-reporting is zero.


(3) $$P_{m}⊥\text{Time}$$

This assumption refers to the number of pregnant women (P) in a given municipality (*m*) being independent of the time analyzed, for example, that this number does not change significantly at any time of the year. We considered the assumption valid given the short period assessed (six months).

### Procedures

To calculate under-reporting by municipality based on the pregnant population, it was necessary to estimate the incidence of dengue in this population during the study period. For this, we obtained an estimate of the average number of pregnant women aged 18-45 years per municipality in the first half of 2024 using the following equation:

*Estimated number of pregnant women = live births in the period × GD*

Where the number of live births refers to those to mothers aged 18-45 years; and *GD* corresponds to the mean gestational duration expressed in units of the study period, calculated as the ratio between the mean gestational duration (in weeks) and the number of weeks in the analyzed period. For example, if the average gestational duration recorded in the semester was 39 weeks, we divide this value by the 26 weeks corresponding to the semester (the duration of the observation period), yielding GD = 1.5 semesters.

Thus, the number of live births was used as a proxy for the flow of pregnancies, adjusted by the mean gestational duration in the semester, analogous to the epidemiological principle that prevalence = incidence × duration, under approximately stable conditions^22^. In the supplementary material, we include a validation of the formula.

We compared our estimates with those from the method adopted by the Ministry of Health using Spearman’s rank correlation coefficient. In the Ministry’s calculation, proposed to municipalities for estimating the number of pregnant women and allocating related resources, the number of live births in the period is increased by 10% to compensate for losses due to miscarriage or under-reporting^23,24^, but without adjustment for gestational duration.

The Ministry of Health’s method estimates only the number of pregnancies that occurred over the year, whereas the aim of this study was to estimate the average number of pregnant women at any point during the period. Considering that the mean duration of pregnancy is longer than the study time window, using the number of live births directly as the denominator would tend to underestimate the effective exposure time of pregnant women and, consequently, overestimate dengue incidence. The equation sought to adjust the denominator to the risk period assessed, providing a more appropriate estimate of the pregnant population at risk. We acknowledge that misclassification errors, such as under-reporting in pregnant women (numerator) and losses due to pregnancies ending early (denominator), may introduce distortions in the estimates, but we assume that these biases are small, act in opposite directions and would tend to offset one another.

Next, we calculated the dengue incidence rate in pregnant women as the ratio between the number of dengue cases identified in pregnant women and the estimated number of pregnant women, assuming this estimate remained constant over the period. To obtain the incidence in the non-pregnant group, the numerator was the number of dengue cases in non-pregnant women, and the denominator was the total population of women in this age group in the municipality, minus the estimated number of pregnant women.

Therefore, for every municipality showing *D_p_ ≥ D_np_,* we calculated the Under-reporting Proportion (UP) with the following equation:


$$UP=\frac{D_{p}-D_{np}}{D_{p}}$$

A positive result (UP > 0) was interpreted as indicating that the municipality had under-reporting in non-pregnant women compared with pregnant women. In municipalities with *D_p_ < D_np_* UP was set to zero, assuming that sensitivity in non-pregnant women could not be higher than in pregnant women.

Based on the estimated under-reporting in the population of non-pregnant women and the number of PC in this group *(PC_np_),* we calculated the number of undetected cases per municipality using the following equation:


$$Undetected\;cases=\frac{PC_{np}}{\left(1-UP\right)}-PC_{np}$$

Subsequently, using the municipal-level data obtained, we carried out statistical analyses to explore the association with potential determinants of under-reporting. In this case, the dichotomous dependent variable was positive versus null under-reporting, and the independent variables were: *ESF coverage, number of primary health units (UBS) per thousand women of reproductive age (WRA), and degree of urbanization*, assuming that these factors could influence access to the health system for the population of interest. Estimates were obtained using logistic regression, with odds ratios (OR) as measures of association and a significance level of 0.05. Analyses were performed in STATA, version 18.0.

### Spatial autocorrelation analysis

Additionally, the results obtained from the under-reporting estimates were processed in spatial analysis software to visualize the distribution of municipalities with positive under-reporting and to identify spatial dependence between these municipalities. A queen-type spatial weights matrix was used for the autocorrelation analysis. The univariate Global Moran’s I was used to measure the overall spatial association in the dataset, and the Local Indicator of Spatial Association (LISA) was used to produce the Moran scatterplot, the LISA cluster map and the significance map, using a pseudo-significance test with 999 permutations for validation.

In addition, we used a spatial lag model to assess residual spatial dependence and possible associations between the explanatory variables and the under-reporting rate in non-pregnant women, defined as the ratio between the estimated number of unidentified cases and the population of non-pregnant women. The software used was GeoDa, version 1.22.

#### Data Availability Statement:

The full dataset supporting the findings of this study has been made available in SciELO Data and can be accessed at https://doi.org/10.48331/SCIELODATA.LKQZDT.

## RESULTS

Between January and June 2024, the State of São Paulo recorded 2,044,014 probable dengue cases^1^. Of these, 1,104,635 (54%) occurred in women, of which 14,674 (1.33%) were recorded in pregnant women. In the 18–45-year age group, 83.1% of pregnant women’s cases were concentrated (n = 12,201). Cases in non-pregnant women aged 18-45 years totaled 506,471.

The estimates of pregnant women per municipality obtained using the proposed equation, when compared with the estimates from the method adopted by the Ministry of Health for the same period, yielded a Spearman correlation coefficient of 0.99 (p < 0.001), indicating a strong correlation between the methods. However, the values estimated by our method were on average 33% higher than those obtained using the Ministry’s method.

The distribution of under-reporting percentages by municipality was classified according to Table 1, where we observed that most municipalities (83.3%) did not present positive under-reporting, while 108 municipalities (16.7%) showed values ranging from 1% to 95%.

**Table 1:** Distribution of municipalities by estimated percentage of dengue under-reporting in the first half of 2024, State of São Paulo, ESP.

Estimated under-reporting percentage	n	%	Accumulated %
0	537	83.3	83.3
1 |--| 25	41	6.4	89.7
26 |--| 50	27	4.2	93.8
51 |--| 75	24	3.7	97.6
76 |--| 100	16	2.5	100
**Total**	645	100	-

**Figure 1:**
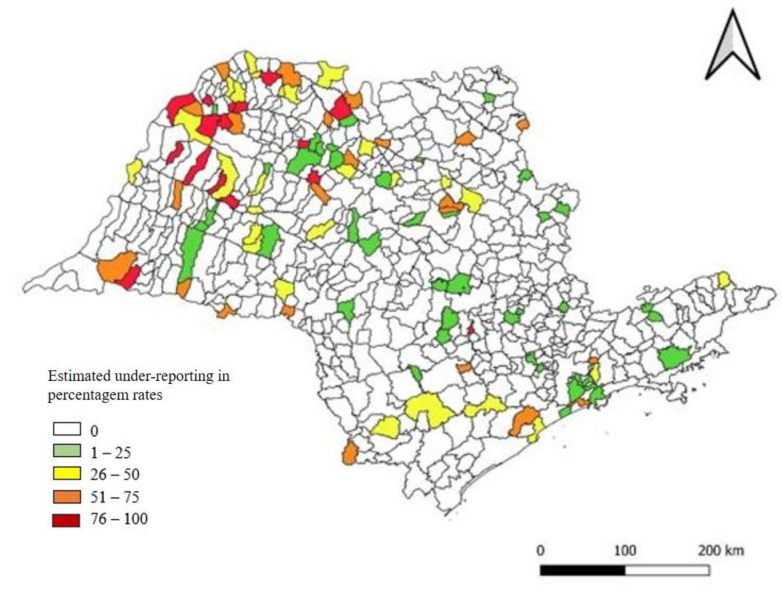
Spatial distribution of municipalities classified according to estimated under-reporting percentage, ESP, 2024.

Source: elaborated with the GeoDA 1.22 software.

The map (Figure 1) shows the spatial distribution of municipalities according to the estimated under-reporting percentage, where we observe a concentration of municipalities with under-reporting above 50% in the north-western region of the state and a heterogeneous distribution in the other regions.

From these estimates, we calculated that there would be 8,624 probable dengue cases undetected in non-pregnant women aged 18-45 years in the period, which would represent a 0.42% increase in the total number of probable cases recorded in the state.

The independent variables showed wide variation between municipalities: the degree of urbanization ranged from 25.9% to 100% (M = 87.3; SD = 13.2), ESF coverage ranged from 6% to 436% potential population coverage (M = 120; SD = 54.4), and the number of primary health units per thousand WRA ranged from 0 to 12 (M = 1.27; SD = 1.05), indicating marked heterogeneity in terms of urban structure and primary care. In the regression analyses, we did not identify any significant association between positive under-reporting and the variables assessed (Table 2).

**Table 2:** Logistic regression analysis of independent variables in relation to positive under-reporting, ESP 2024.

Positive under-reporting	*Odds ratios*	P-value	95%CI
ESF coverage^*^	1.37	0.08	0.96–1.99
UBS per WRA	1.1	0.15	0.95–1.3
Degree of urbanization	0.99	0.37	0.98–1

*Analyzed as a continuous variable, with the association referring to a change from 0 to 100% coverage.

Source: calculations based on the under-reporting estimates and data from Tabnet SES-SP (2024), Ministry of Health (2024) and Fundação SEADE-SP (2024).

**Figure 2:**
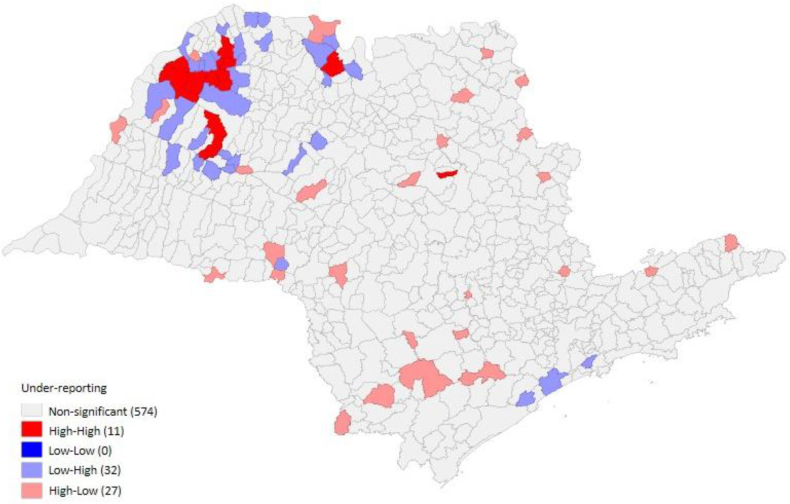
Moran cluster map with classification of municipalities by level of spatial autocorrelation, according to under-reporting percentage, ESP, 2024.

Source: elaborated with the GeoDA 1.22 software.

**Figure 3:**
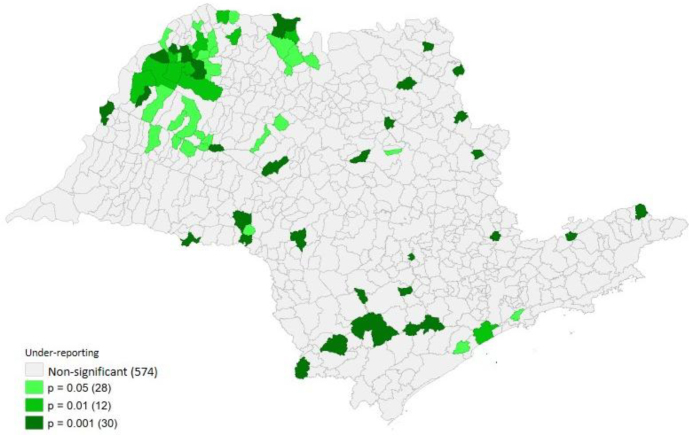
Moran significance map with classification of municipalities by level of statistical significance in the spatial analysis, according to under-reporting percentage, ESP, 2024.

Source: elaborated with the GeoDA 1.22 software.

The spatial autocorrelation analysis identified clusters of municipalities with positive under-reporting values and neighboring areas with similar values (Figures 2 and 3). The municipality of Ilhabela was removed from the spatial analysis because, as an island, it has no territorial contiguity with other municipalities.

**Figure 4:**
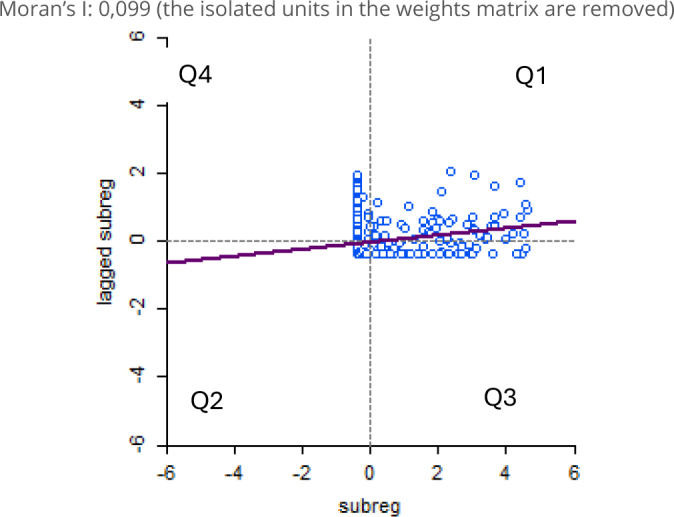
Moranscatterplotbymunicipalunder-reporting percentage, ESP, 2024.

Source: elaborated with the GeoDA 1.22 software.

The Moran scatter plot (Figure 4) showed a high-high (Q1) pattern among municipalities with positive under-reporting and similar neighbors. There were no points in the other quadrants, as negative values were not considered. The value of the Global Moran’s I index was 0.099 (p = 0.001), indicating positive but weak spatial correlation.

The Moran cluster map (Figure 2) showed 11 municipalities (high-high), clustered in the north-western region of the state, with under-reporting and similar neighboring municipalities; 32 municipalities (5%) showed a low-high correlation, with no positive values themselves but neighboring municipalities with under-reporting. In 27 municipalities (4.2%) the correlation was high-low, where they presented positive mean values but their neighbors did not. In 574 municipalities (89%) there was no significant spatial correlation. The significance map (Figure 4) showed 70 municipalities (p < 0.05) with statistically significant results for the observed pattern.

The spatial lag model yielded ρ = 0.07 (p = 0.26), without statistical significance, indicating no residual spatial dependence after adjusting for covariates (Table 3). However, we observed a significant positive association between ESF coverage and the rate of undetected cases in non-pregnant women (β = 3.26; p = 0.01), suggesting that municipalities with higher ESF coverage tend to present higher estimated under-reporting in this group.

**Table 3:** Results of the spatial lag regression model and variables associated with the dengue under-reporting rate in non-pregnant women, ESP, 2024.

Variable	Coefficient	Standard-error	Z	P
Spatial lag coefficient (ρ)	0.07	0.062	1.14	0.26
UBS per one thousand WRA	1.23	0.65	1.9	0.06
ESF coverage	3.26	1.27	2.56	0.01
Degree of urbanization	-0.003	0.046	-0.07	0.94
Constant	-1.71	4.71	-0.36	0.71

Source: calculations based on the under-reporting estimates. Data: Tabnet SES-SP (2024), Ministry of Health (2024) and Fundação Seade-SP (2024).

## DISCUSSION

The results show that most municipalities (83.3%) did not present evidence of under-reporting of probable dengue cases in non-pregnant women aged 18-45 years compared with pregnant women. This suggests good sensitivity of the surveillance system in recording suspected dengue cases. However, under-reporting may have occurred in both groups, which would not be detected by this methodology, since this strategy depends on the coverage and quality of antenatal care, which may vary widely between municipalities. Conversely, in municipalities with positive under-reporting, we interpret that there was lower sensitivity of the health system to identify and notify suspected dengue cases in the non-pregnant population, considering the study’s assumptions.

Despite the lack of a significant association between under-reporting and variables that would intuitively be related, this result may have several explanations. The short analysis period and wide geographic scope may have been insufficient to reveal the effects of the determinants of under-reporting. In addition, socioeconomic inequalities, cultural and behavioral factors related to care-seeking, and local epidemiological characteristics were not considered in this study. Nevertheless, we believe that the proposed methodology can be applied on a larger scale to explore other potential determinants of under-reporting and to monitor interventions aimed at improving the sensitivity of the surveillance system.

The spatial autocorrelation analysis revealed spatial dependence among some municipalities in the north-western region of the state, possibly influenced by variables not considered in this study. Although the spatial lag model did not identify residual spatial dependence between municipalities, we observed a positive and significant association between ESF coverage and the rate of undetected cases in non-pregnant women. Although this may seem counterintuitive, one hypothesis is that ESF coverage increases detection capacity mainly among pregnant women. This would indirectly highlight under-reporting in non-pregnant women. However, this requires further investigation with a causal-inference focus, using appropriate models that account for spatial clustering patterns. In addition, under-reporting, which was analyzed here as a dichotomous variable, could also be quantified and analyzed as a count outcome.

Although the causes of under-reporting are not yet fully elucidated, studies indicate among its determinants the lack of knowledge among health professionals, infrastructure deficiencies, failures in completing notification forms and prioritization of severe cases or epidemic periods^3,10,25^. Mild or oligosymptomatic dengue cases tend to be less frequently reported, either because patients do not seek medical care or because of the absence of laboratory diagnosis in patients with non-specific symptoms^25,26^.

Some studies use statistical methods to adjust for under-reporting, applying models that link the expected spatial distribution of dengue and estimates of the probability of virus transmission in a given population, combined with expansion factors^27^. Although useful, these approaches are constrained by determinants that depend on the epidemiological situation, viral circulation and climatic factors, which vary across regions. They may therefore have important gaps.

Other studies use an expansion factor, that is, a rate by which the recorded number ofcases is multiplied, based on cohort studies and reviews of outpatient and hospital records to correct for under-reporting^2,26,28,29^. Although widely used, this approach depends on access to these data, on accurate and complete recording of patient care, and on laboratory diagnosis.

Our approach has the advantage of using as a reference a group that is expected to receive the best possible care, due to pregnancy, in a country that promotes universal access and is therefore presumed to offer broad access to the best diagnostic resources. However, studies indicate significant variations in coverage, quality of antenatal care and access to diagnostic services between municipalities in the State of São Paulo^30,31,32,33^. These findings indicate that the effectiveness of care for pregnant women varies locally and may affect the sensitivity of methodologies that use this group as a reference to estimate under-reporting. Regional inequalities in notification quality also persist, which may compromise the accuracy of epidemiological analyses^34^.

Despite its novel approach, this study is subject to limitations inherent to the ecological design, such as ecological fallacy, which prevents individual-level inferences based on aggregated data and requires caution in interpreting the findings. In addition, municipal heterogeneity, unmeasured confounding factors and the quality of secondary data may affect the assessment of associations. In Sinan, we found missing or inconsistent data that ultimately raise doubts about data selection for analysis. An example is 721 records of dengue cases in pregnant women aged over 60 years, a group well beyond reproductive age.

The proposed methodology has the potential to be applied in other epidemiological contexts, especially for notifiable diseases whose incidence is expected to be similar between pregnant and non-pregnant women, such as dengue, where pregnancy is expected to lead to greater use of health services. Importantly, this approach can be applied on a large scale, using routinely available data from health information systems. However, its applicability also depends on specific conditions, such as the availability of reliable data on pregnant women and adequate antenatal care coverage. Another important point is that this method cannot be used for all notifiable diseases. Specifically, in situations where pregnancy increases the risk of acquiring or developing the event – for example, chronic diseases such as diabetes and hypertension – this method could not be applied, as it would violate the assumption of similar risk between pregnant and non-pregnant women.

In conclusion, we present a methodology to identify and estimate under-reporting of dengue cases using incidence in pregnant women as a reference. Applying the method to municipalities in the State of São Paulo made it possible to identify a set of municipalities with evidence of under-reporting, concentrated mainly in one region of the state. The approach showed potential to support the identification of determinants of under-reporting and to guide improvements in epidemiological surveillance. Although we explored some possible determinants in this study, applying the method in different epidemiological contexts, with different variables and for other notifiable diseases should be the focus of future investigations.
