# Supplementary material for: Pregnant women as a reference population to estimate dengue under-reporting: methodological proposal and application in São Paulo State, Brazil
Source: Rev Bras Epidemiol. 2026 Jul 27;29:e260023. doi: 10.1590/1980-549720260023 (PMC13405553; doi:10.1590/1980-549720260023)
Supplement: Translation

# Translation: Gestantes como população de referência para estimar a subnotificação de dengue: proposta metodológica e aplicação no estado de São Paulo, Brasil

**DOI:** 10.1590/1980-549720260023.2

**Authors:** Leandro Cesar Pompeu, Fredi Alexander Diaz-Quijano

**Affiliations:** I Universidade de São Paulo, Public Health School, Postgraduate Programme in Public Health Entomology – São Paulo (SP), Brasil.; II Universidade de São Paulo, Public Health School, Department of Epidemiology, Laboratory of Causal Inference in Epidemiology – São Paulo (SP), Brasil.

**Keywords:** Dengue, Subnotificação, Vigilância epidemiológica, Gestantes, Saúde pública

## Abstract

**Objetivo:**

Propor e aplicar uma metodologia para estimar a subnotificação de casos prováveis de dengue em mulheres não gestantes, utilizando a incidência em gestantes como referência.

**Métodos:**

Estudo ecológico com dados de casos prováveis de dengue notificados no estado de São Paulo entre janeiro e junho de 2024. Partindo do pressuposto de que gestantes apresentam maior probabilidade de diagnóstico e notificação devido ao acompanhamento pré-natal, tomamos esse grupo como referência para calcular a subnotificação da incidência de dengue em não gestantes na faixa etária de 18 a 45 anos. A associação entre a subnotificação e os fatores “cobertura da Estratégia Saúde da Família”, “número de Unidades Básicas de Saúde por mil mulheres em idade fértil” e “grau de urbanização” foi avaliada por regressão logística. Também foi realizada análise de autocorrelação espacial da subnotificação.

**Resultados:**

Dos 645 municípios analisados, 108 (16,7%) foram sugestivos de subnotificação, variando de 1 a 95%. Estimou-se que 8.624 casos de dengue não tenham sido detectados em mulheres não gestantes no período. Não identificamos associações estatisticamente significativas entre os fatores analisados e a subnotificação. A análise espacial indicou agrupamentos significativos de municípios com subnotificação na região noroeste do estado.

**Conclusão:**

A metodologia proposta pode identificar áreas com provável subnotificação e subsidiar análises sobre determinantes da sensibilidade do sistema de vigilância, contribuindo para orientar intervenções. Embora potencialmente aplicável a outras doenças de notificação compulsória, comparações entre gestantes e não gestantes devem ser interpretadas com cautela, devido a diferenças de risco, vulnerabilidade e critérios diagnósticos.

## INTRODUÇÃO

A dengue é a arbovirose mais importante no mundo. Em 2024, o Brasil registrou incidência recorde, com 6.636.763 casos prováveis notificados até dezembro do referido ano^1^. Além disso, a subnotificação de casos é esperada na vigilância da dengue, principalmente devido à elevada proporção de infecções leves ou assintomáticas, estimadas em cerca de 75% dos casos^2^. Apesar de ser um problema reconhecido globalmente, a subnotificação permanece de difícil identificação e quantificação^3^, podendo levar à subestimação da real carga da doença e dificultar o controle e a prevenção^4^.

As gestantes estão entre os grupos de risco para dengue grave, e estudos indicam associação com maior risco de parto prematuro e morte fetal^5,6^. Por isso, é razoável supor que o atendimento à mulher gestante apresenta maior sensibilidade na identificação de doenças, sustentada por fatores como acompanhamento pré-natal frequente, atenção especializada e rotina de monitoramento mais rigorosa^7,8,9^. Assim, hipotetizamos que a estimativa da incidência de casos em gestantes reflete de maneira mais próxima a incidência real da doença, servindo como referência para estimar a subnotificação em outros grupos populacionais com risco similar.

Embora existam diversas estratégias para estimar a subnotificação, incluindo buscas ativas em serviços de saúde, inquéritos populacionais e projeções baseadas em dados indiretos^10,11^, não identificamos estudos que explorem o uso de grupos específicos com maior sensibilidade para detecção de casos como referência para essa estimativa. O uso de tais grupos poderia facilitar a quantificação da subnotificação em grande escala, permitindo identificar padrões de agregação e analisar a influência de seus determinantes.

Em consequência, propomos e aplicamos uma metodologia para estimar a subnotificação em mulheres não gestantes utilizando como referência a incidência de dengue em gestantes. Dessa forma, identificamos municípios com possível subnotificação e avaliamos fatores potencialmente associados. Este método teria potencial para ajudar a implementar e acompanhar estratégias para melhorar a sensibilidade do sistema de vigilância epidemiológica.

## MÉTODOS

Trata-se de um estudo ecológico utilizando dados públicos e anonimizados dos sistemas do governo federal e estadual, de notificações de Casos Prováveis (CP) de dengue, que correspondem aos casos notificados menos os descartados, em mulheres dos municípios do Estado de São Paulo (ESP) entre janeiro e junho de 2024. Os dados foram extraídos em janeiro de 2025.

Embora a faixa etária usualmente considerada como idade fértil seja de 10 a 49 anos^12^, neste estudo analisamos apenas as incidências em gestantes e não gestantes de 18 a 45 anos. A faixa etária foi escolhida para garantir a comparabilidade entre os grupos quanto ao risco de infecção e comportamento de busca por serviços de saúde, além de permitir excluir faixas etárias mais extremas, nas quais podem ocorrer diferenças de exposição, diagnóstico e atenção recebida. Mulheres não gestantes abaixo de 18 anos apresentam padrões distintos de utilização dos serviços de saúde, geralmente com menor procura por atendimento^13,14,15,16^, enquanto gestantes adolescentes costumam receber acompanhamento intensificado durante o pré-natal^13,14,17,18^. Assim, esta restrição visou minimizar vieses de seleção e tornar os grupos mais homogêneos quanto à exposição ao vírus e à sensibilidade da vigilância para detecção dos casos.

### Fontes de dados

Os CP de dengue foram extraídos do Sistema de Informações de Agravos de Notificação (Sinan), disponível no Departamento de Informação e Informática do Sistema Único de Saúde (DataSUS), e tabulados por meio do software *Tabwin*, versão 4.15^1^. Estes dados incluíram a situação de gestante como uma das variáveis coletadas sistematicamente, o que permitiu ter os numeradores para calcular incidências em gestantes e não gestantes.

Os denominadores para calcular as taxas de incidências foram deduzidos a partir de uma projeção do número de nascidos vivos por município no período, os quais foram extraídos do Sistema de Informação sobre Nascidos Vivos (Sinasc), disponível no Portal Brasileiro de Dados Abertos^19^.

Dados populacionais e as variáveis grau de urbanização e número de Unidades Básicas de Saúde (UBS) por município, atualizados até junho de 2024, foram extraídos do Tabnet da Secretaria Estadual de Saúde de São Paulo (SESSP) e das estimativas do Sistema Estadual de Análise de Dados (Seade) para 2023, com base no Censo de 2022^20^. Os dados de cobertura da Estratégia Saúde da Família (ESF) foram obtidos no site e-Gestor Atenção Básica do Ministério da Saúde (MS), com base na cobertura potencial que contempla as equipes financiadas com recursos federais, estaduais e municipais^21^.

### Pressupostos

Para este estudo, adotamos os seguintes pressupostos para o método proposto:


(1) $$I_{g}=I_{ng}|município,idad$$

Esta equação se refere à assunção de que a incidência de dengue em gestantes *(I_g_*) é igual à incidência em não gestantes *(I_ng_*), condicionada a fatores como município e idade. Em relação a esta última variável, assumimos que a restrição ao grupo de estudo seria um condicionamento suficiente, como já mencionado.


(2) $$D_{g}\geq D_{ng}|município,idad$$

Nesta equação, assumimos que a incidência detectada em gestantes *(D_g_*) deve ser igual ou maior do que a detectada em não gestantes *(D_ng_),* quando controladas pelas variáveis município e idade, pois gestantes tendem a ter maior contato com o sistema de saúde, o que torna mais provável que casos leves ou moderados de dengue sejam detectados e notificados. Por isso, é esperado que *D_g_* ≥ *D_ng_* mesmo que a incidência real seja igual em ambos os grupos. Partindo do anterior, consideramos que em situações onde *D_g_ < D_ng_* a subnotificação seria igual a zero.


(3) $$G_{m}⊥\text{Tempo}$$

Este suposto se refere a que o número de gestantes (G) em um determinado município (*m*) é independente do tempo analisado, ou seja, que este número não muda significativamente em qualquer momento do ano. Consideramos o suposto válido dado o curto período avaliado (seis meses).

### Procedimentos

Para calcular a subnotificação por município com base na população de gestantes, foi necessário estimar a incidência de dengue nesta população no período de estudo. Para isso, obtivemos uma estimativa do número médio de gestantes, de 18 a 45 anos, por município no 1º semestre de 2024 com a seguinte equação:

*Estimativa de gestantes = nascidos vivos no período × DG*

Onde, o número de nascidos vivos refere-se àqueles de mães com idade entre 18 e 45 anos; e *DG* corresponde à duração gestacional média em unidades do período de estudo, calculado pela razão entre a duração gestacional média (em semanas) e o número de semanas do período analisado. Por exemplo, se a média da duração gestacional registrada no semestre foi de 39 semanas, dividimos esse valor pelas 26 semanas correspondentes ao semestre (que é a duração do período de observação), resultando no valor de DG = 1,5 semestre.

Assim, o número de nascidos vivos foi utilizado como proxy do fluxo de gestações ajustado pela duração média da gestação no semestre, em analogia ao princípio epidemiológico de que prevalência = incidência × duração, sob condições aproximadamente estáveis^22^. No material suplementar, incluímos uma validação da fórmula.

Comparamos nossas estimativas com as estimativas do método adotado pelo Ministério da Saúde utilizando o coeficiente de correlação de Spearman. No cálculo do MS, proposto aos municípios para estimativa do número de gestantes e destinar recursos relacionados, utiliza-se o número de nascidos vivos no período acrescido de 10%, para compensar perdas por aborto ou subnotificação^23,24^, porém sem o ajuste pela duração gestacional.

O método do MS estima somente o número de gestações que ocorreram ao longo do ano, enquanto que o propósito neste estudo foi estimar o número médio de gestantes em qualquer momento do período. Considerando que a duração média da gestação é superior à janela temporal do estudo, o uso direto do número de nascidos vivos como denominador tenderia a subestimar o tempo efetivo de exposição das gestantes e, consequentemente, superestimar a incidência da dengue. A equação buscou ajustar o denominador ao período de risco avaliado, representando uma estimativa mais adequada da população gestante exposta. Reconhecemos que erros de classificação, como subnotificação em gestantes (numerador) e perdas por gestações interrompidas precocemente (denominador), podem introduzir distorções nas estimativas, mas assumimos que esses vieses são pequenos, atuam em direções opostas e tenderiam a se compensar.

Em seguida, calculamos a taxa de incidência de dengue em gestantes por meio da razão entre o número de casos de dengue identificados em gestantes e a estimativa do número de gestantes, assumindo que esta estimativa se manteria constante ao longo do período. Para obter a incidência no grupo das não gestantes, o numerador foi o número de casos de dengue em mulheres não gestantes, e o denominador foi a população total deste grupo de idade no município, subtraindo a estimativa de gestantes.

Dessa forma, para todo município com *D_g_* ≥ *D_ng_*, calculamos a Proporção de Subnotificação (PS) com a seguinte equação:


$$PS=\frac{D_{g}-D_{ng}}{D_{g}}$$

Um resultado positivo (PS > 0) foi interpretado como indicador de que o município teve subnotificação em mulheres não gestantes em relação às gestantes. Em municípios com *D_g_ < D_ng_* a PS foi considerada igual a zero, assumindo que a sensibilidade em não gestantes não poderia ser superior à sensibilidade em gestantes.

Com base na estimativa de subnotificação na população de mulheres não gestantes e da quantidade de CP neste grupo *(CP_ng_*), calculamos o número de casos não detectados por município pela seguinte equação:


$$Casos\;não\;detectados=\frac{CP_{ng}}{\left(1-PS\right)}-CP_{ng}$$

Posteriormente, com os dados obtidos por município, realizamos análises estatísticas para explorar associação com possíveis fatores determinantes de subnotificação. Nesse caso, como variável dependente dicotômica tomamos a subnotificação positiva versus nula, e como variáveis independentes: *cobertura de ESF*, *número de UBS por mil Mulheres em Idade Fértil (MIF)* e *grau de urbanização*, assumindo que estes fatores poderiam influenciar o acesso ao sistema de saúde pela população de interesse. As estimativas foram obtidas usando a regressão logística, calculando *Odds Ratios* (OR) como medidas de associação, com nível de significância de 0,05. As análises foram realizadas no *STATA*, versão 18.0.

### Análise de autocorrelação espacial

Adicionalmente, os resultados obtidos da estimativa de subnotificação foram geoprocessados num software de análise espacial para visualizar a distribuição dos municípios com subnotificação positiva e identificar dependência espacial entre esses municípios. A matriz de pesos utilizada para a análise de autocorrelação foi do tipo queen. O Índice de Moran Global univariado foi utilizado para medir a associação espacial existente no conjunto dos dados geral; e o Indicador Local de Associação Espacial (LISA), para produzir o diagrama de espelhamento de Moran, o LISA Map (mapa de agrupamento) e o mapa de significância, utilizando para validação o teste de pseudo-significância com 999 permutações.

Além disso, utilizamos um modelo de defasagem espacial (*Spatial Lag Model*) para verificar dependência espacial residual e possíveis associações entre as variáveis explicativas e a taxa de subnotificação em mulheres não gestantes, definida como a razão entre a estimativa de casos não identificados e a população de mulheres não gestantes. O software utilizado foi o GeoDa, versão 1.22.

#### Declaração de Disponibilidade de Dados:

Todo o conjunto de dados que dá suporte aos resultados deste estudo foi disponibilizado no SciELO Data e pode ser acessado em https://doi.org/10.48331/SCIELODATA.LKQZDT.

### RESULTADOS

Entre janeiro e junho de 2024, o ESP registrou 2.044.014 casos prováveis de dengue^1^. Desses, 1.104.635 (54%) ocorreram em mulheres, dos quais 14.674 (1,33%) foram registrados em gestantes. Na faixa etária de 18 a 45 anos, concentraram-se 83,1% dos casos de gestantes (n=12.201). Os casos em mulheres não gestantes com idade entre 18 e 45 anos totalizaram 506.471.

As estimativas de gestantes por município, obtidas da equação proposta, quando comparadas às estimativas do método adotado pelo Ministério da Saúde para o mesmo período, resultaram num coeficiente de correlação de Spearman de 0,99 (p<0,001), indicando forte correlação entre os métodos. No entanto, os valores estimados pelo nosso método foram, em média, 33% superiores aos obtidos pelo método do MS.

A distribuição dos percentuais de subnotificação por município foi classificada de acordo com a Tabela 1, onde observamos que a maioria dos municípios (83,3%) não apresentou subnotificação positiva, enquanto 108 municípios (16,7%) mostraram valores variando de 1% a 95%.

**Tabela 1:** Distribuição dos municípios por percentual de subnotificação estimada de casos de dengue no primeiro semestre de 2024, ESP.

Porcentagem de subnotificação estimada	n	%	% acumulada
0	537	83,3	83,3
1 |--| 25	41	6,4	89,7
26 |--| 50	27	4,2	93,8
51 |--| 75	24	3,7	97,6
76 |--| 100	16	2,5	100
**Total**	645	100	-

O mapa (Figura 1) apresenta a distribuição espacial dos municípios de acordo com o percentual de subnotificação estimada, onde observamos uma concentração de municípios com subnotificação acima de 50% na região noroeste do estado e distribuição heterogênea nas demais regiões.

**Figura 1:** Distribuição espacial dos municípios classificados segundo percentual de subnotificação estimada, ESP, 2024.

Fonte: elaborada com o *software* GeoDA 1.22.

A partir das estimativas, calculamos que haveria 8.624 casos prováveis de dengue não detectados em mulheres não gestantes de 18 a 45 anos no período, o que representaria um aumento de 0,42% no total de casos prováveis registrados no estado.

As variáveis independentes apresentaram ampla variação entre os municípios: o grau de urbanização variou de 25,9 a 100% (M=87,3; DP=13,2), a cobertura de ESF variou de 6 a 436% de cobertura potencial da população (M=120; DP=54,4) e o número de UBS para cada mil MIF variou de 0 a 12 (M=1,27; DP=1,05) – indicando forte heterogeneidade em termos de estrutura urbana e de atenção básica. Nas análises de regressão, não identificamos associação significativa entre a subnotificação positiva e as variáveis avaliadas (Tabela 2).

**Tabela 2:** Análise de regressão logística das variáveis independentes em relação à subnotificação positiva, ESP 2024.

Subnotificação positiva	*Odds ratios*	Valor p	IC95%
Cobertura de ESF^*^	1,37	0,08	0,96–1,99
UBS por mil MIF	1,1	0,15	0,95–1,3
Grau de urbanização	0,99	0,37	0,98–1

*Analisada como variável contínua, sendo a associação relativa a uma mudança de 0 a 100% de cobertura.

Fonte: cálculos realizados com base na estimativa de subnotificação e em dados do Tabnet SES-SP (2024), Ministério da Saúde (2024) e da Fundação SEADE-SP (2024).

A análise de autocorrelação espacial identificou *clusters* de municípios com valores positivos de subnotificação e vizinhos com valores semelhantes (Figuras 2 e 3). O município de Ilhabela foi removido da análise espacial por se tratar de uma ilha e não possuir relação de vizinhança territorial.

**Figura 2:** Mapa de agrupamento de Moran com classificação dos municípios por nível de autocorrelação espacial, segundo percentual de subnotificação, ESP, 2024.

Fonte: elaborada com o *software* GeoDA 1.22.

**Figura 3:** Mapa de significância de Moran com a classificação dos municípios por nível de significância estatística da análise espacial, segundo percentual de subnotificação, ESP, 2024.

Fonte: elaborada com o *software* GeoDA 1.22.

O Diagrama de Espalhamento de Moran (Figura 4) mostrou padrão alto/alto (Q1) entre municípios com sub-notificação positiva e vizinhos semelhantes. Nos demais quadrantes não houve pontos, pois valores negativos não foram considerados. O valor do Índice de Moran Global foi de 0,099 (p=0,001), o que indicou correlação espacial positiva, porém fraca.

**Figura 4:** Diagrama de Espalhamento de Moran segundo percentual de subnotificação por município, ESP, 2024.

*****

O Mapa de Agrupamento de Moran (Figura 2) apresentou 11 municípios (alto/alto), aglomerados na região noroeste do estado com subnotificação e seus vizinhos semelhantes; 32 municípios (5%) apresentaram correlação baixo-alto, sem valores positivos, mas seus vizinhos apresentaram subnotificação. Em 27 municípios (4,2%) a correlação foi do tipo alto-baixo, onde apresentaram valores médios positivos, mas seus vizinhos não. Em 574 municípios (89%) não houve correlação espacial significativa. O mapa de significância (Figura 4) apresentou 70 municípios (p<0,05) com significância estatística para os resultados observados.

O modelo de defasagem espacial apresentou ρ=0,07 (p=0,26), sem significância estatística, indicando ausência de dependência espacial residual após o ajuste das covariáveis (Tabela 3). No entanto, observamos associação significativa e positiva entre cobertura de ESF e taxa de casos não detectados em não gestantes (β=3,26; p=0,01), sugerindo que municípios com maior cobertura de ESF tendem a apresentar maior subnotificação estimada nesse grupo.

**Tabela 3:** Resultados do modelo de regressão espacial do tipo defasagem (*Spatial Lag Model*) e variáveis associadas à taxa de subnotificação de dengue em mulheres não gestantes, ESP, 2024.

Variável	Coeficiente	Erro-padrão	Z	P
Coeficiente de defasagem espacial (ρ)	0,07	0,062	1,14	0,26
UBS por mil MIF	1,23	0,65	1,9	0,06
Cobertura de ESF	3,26	1,27	2,56	0,01
Grau de urbanização	-0,003	0,046	-0,07	0,94
Constante	-1,71	4,71	-0,36	0,71

Fonte: cálculos realizados com base na estimativa de subnotificação. Dados: Tabnet SES-SP (2024), Ministério da Saúde (2024) e Fundação SEADE-SP (2024).

## DISCUSSÃO

Os resultados demonstram que a maioria dos municípios (83,3%) não apresentou evidências de subnotificação de casos prováveis de dengue no grupo de mulheres não gestantes de 18 a 45 anos em relação às gestantes. Isso sugere boa sensibilidade do sistema de vigilância no registro de casos suspeitos de dengue. No entanto, é possível que tenha ocorrido subnotificação em ambos os grupos, o que não seria detectado por esta metodologia, já que essa estratégia depende da cobertura e da qualidade da assistência pré-natal, que podem variar amplamente entre municípios. De outro modo, nos municípios com subnotificação positiva, interpretamos que houve menor sensibilidade do sistema de saúde para identificar e notificar casos suspeitos de dengue na população não gestante, considerando os pressupostos do estudo.

Apesar da ausência de associação significativa entre subnotificação e variáveis que intuitivamente estariam relacionadas, esse resultado pode ter várias explicações. O período curto de análise e a abrangência geográfica podem ter sido insuficientes para evidenciar os efeitos dos determinantes da subnotificação. Além disso, desigualdades socioeconômicas, fatores culturais e comportamentais relacionados à procura por atendimento, e características epidemiológicas locais não foram consideradas neste estudo. No entanto, acreditamos que a metodologia proposta pode ser utilizada em uma escala maior para explorar outros potenciais determinantes de subnotificação e monitorar intervenções para melhorar a sensibilidade do sistema de vigilância.

A análise de autocorrelação espacial revelou dependência espacial entre alguns municípios da região noroeste do estado, possivelmente influenciada por variáveis não consideradas neste estudo. Embora o modelo de defasagem espacial não tenha identificado dependência espacial residual entre os municípios, observamos associação positiva e significativa entre a cobertura de ESF e a taxa de casos não detectados em não gestantes. Apesar de parecer contraintuitiva, uma hipótese é que a cobertura de ESF aumente a capacidade de detecção principalmente entre gestantes. Isso colocaria em evidência, indiretamente, a subnotificação em não gestantes. Contudo, requer investigações adicionais com propósitos de inferência causal, usando modelos adequados que considerem os padrões de agregação espacial. Além disso, a subnotificação, que aqui foi analisada como variável dicotômica, também poderia ser quantificada e analisada como desfecho de contagem.

Embora as causas da subnotificação ainda não estejam totalmente elucidadas, estudos apontam entre seus determinantes a falta de conhecimento dos profissionais da saúde, deficiências de infraestrutura, falhas no preenchimento da ficha de notificação e priorização de casos graves ou períodos epidêmicos^3,10,25^. Casos leves ou oligossintomáticos de dengue tendem a ser menos notificados, seja pela falta de procura de atendimento médico ou pela ausência de diagnóstico laboratorial em pacientes com sintomas inespecíficos^25,26^.

Alguns estudos utilizam métodos estatísticos para ajustar a subnotificação considerando modelos que associam distribuição espacial esperada da dengue, estimativas da probabilidade da transmissão do vírus numa determinada população, com aferição por fatores de expansão^27^. Embora úteis, são limitados por determinantes que dependem de situação epidemiológica, circulação viral e fatores climáticos que variam de acordo com a região. Portanto, podem ter lacunas importantes.

Outros trabalhos utilizam o fator de expansão, sendo uma taxa pela qual se multiplica o número registrado de casos, baseada em estudos de coorte e revisão de prontuários ambulatoriais e hospitalares para corrigir a subnotificação^2,26,28,29^. Apesar de amplamente utilizado, depende do acesso a estes dados, do registro correto e preciso do atendimento ao paciente e do diagnóstico laboratorial.

Nossa abordagem tem a vantagem de usar como referência um grupo que se espera que tenha o melhor atendimento possível, devido à condição de gestação, em um país onde se promove o acesso universal e, portanto, presume-se que haja amplo acesso aos melhores recursos para diagnóstico. No entanto, estudos apontam variações significativas na cobertura, qualidade do pré-natal e no acesso aos serviços de diagnóstico entre municípios do estado de São Paulo^30,31,32,33^. Esses achados indicam que a efetividade do cuidado à gestante varia localmente e pode afetar a sensibilidade de metodologias que utilizam este grupo como referência para estimar subnotificação. Persistem também desigualdades regionais na qualidade da notificação que podem comprometer a precisão de análises epidemiológicas^34^.

Apesar da abordagem inédita, este estudo está sujeito a limitações inerentes ao delineamento ecológico, como a falácia ecológica, que impede inferências individuais baseadas em dados agregados, exigindo cautela na interpretação dos achados. Além disso, a heterogeneidade dos municípios, fatores de confusão não mensurados e a qualidade dos dados secundários podem impactar na avaliação de associações. No Sinan, encontramos dados ausentes ou discrepantes que acabam gerando dúvidas na seleção dos dados para análise. Exemplo disso são 721 registros de casos de dengue em gestantes com idade acima de 60 anos, faixa etária muito superior à fértil.

A metodologia proposta tem potencial para ser aplicada em outros contextos epidemiológicos, especialmente em doenças de notificação compulsória cuja incidência deve ser similar entre gestantes e não gestantes, como a dengue, em que se espera que a gravidez promova maior procura ao sistema de saúde. De saliente, esta abordagem pode ser aplicada a grande escala, de acordo com dados usualmente disponíveis nos sistemas de informação em saúde. No entanto, sua aplicabilidade também depende de condições específicas, como a disponibilidade de dados confiáveis sobre gestantes e a existência de cobertura adequada de pré-natal. Outro ponto importante é que este método não pode ser utilizado para qualquer doença de notificação. Especificamente, em situações nas quais a gravidez aumenta o risco de adquirir ou desenvolver o evento – por exemplo, doenças crônicas como diabetes e hipertensão arterial – este método não poderia ser aplicado, pois estaria se violando o suposto de risco similar entre gestantes e não gestantes.

Concluindo, apresentamos uma metodologia para identificar e estimar a subnotificação de casos de dengue utilizando a incidência em gestantes como referência. A aplicação do método nos municípios do estado de São Paulo permitiu identificar um conjunto de municípios com indícios de subnotificação, concentrados principalmente numa região do estado. A abordagem demonstrou potencial para apoiar a identificação de determinantes da subnotificação e orientar o aprimoramento da vigilância epidemiológica. Embora tenhamos explorado alguns possíveis determinantes neste trabalho, a aplicação do método em diferentes contextos epidemiológicos, diferentes variáveis e com outras doenças de notificação compulsória deverá ser objeto de investigações futuras.

## COMITÊ DE ÉTICA

Esta pesquisa não requer avaliação por Comitê de Ética em Pesquisa.
